# Evaluating the impact of COVID-19 on rare disease support groups

**DOI:** 10.1186/s13104-021-05579-8

**Published:** 2021-05-06

**Authors:** Julie McMullan, Ashleen L. Crowe, Caitlin Bailie, Amy Jayne McKnight

**Affiliations:** Centre for Public Health, School of Medicine Dentistry and Biomedical Sciences, Institute of Clinical Science Block A, Grosvenor Road, Belfast, BT12 6BA UK

**Keywords:** Charity, Collaborative group, COVID-19, Impact, Online, Qualitative, Rare disease, Survey

## Abstract

**Objectives:**

The impact of the current COVID-19 pandemic has been felt worldwide. Many vulnerable populations rely heavily on peer support provided by individual or collaborative groups. This study aimed to evaluate the impact of COVID-19 on groups supporting patients with a rare disease(s). Anecdotally the current pandemic significantly changed the way in which these groups operate and the services they can provide.

**Data description:**

A targeted survey was conducted online with rare disease individual or collaborative groups. The results of the survey highlight the challenges individual and collaborative groups are facing during this pandemic and help to identify what support should be put in place to enable them to sustain their much-valued service through these trialing times. Groups have experienced an increase in calls to their helplines as well as followers to their websites and social media feeds. Groups are no longer meeting in person and so online meetings, webinars and zoom chats have become a regular occurrence. Fundraising was highlighted as an area of concern for such groups. It is hoped that this data might be used to highlight the support individual and collaborative groups require while also raising awareness of the value they bring to many.

## Objective

Rare diseases are those that affect fewer than 1 in 2000 individuals in the general population [1]. In recent years they have been considered a major public health concern [2]. Although individually rare, they are collectively common, affecting millions of individuals worldwide and putting a huge strain on health services [3]. Those living with a rare disease face many challenges when locating appropriate support [4]. This is largely due to a lack of knowledge and understanding from health and social care professionals, delayed diagnosis and difficulties gaining treatment [5–7].

Previous research has reported that individuals with a rare disease and their families often benefit greatly from peer support which can be found through rare disease individual or collaborative groups (this includes a voluntary group, support group, charity etc.) [8]. The current COVID-19 pandemic has impacted these groups and the services they provide. Due to the low number of responses (n = 6) this data has not been submitted for publication as part of a research paper, but nonetheless offers valuable insights from our rare disease community.

A short online survey was conducted with individual or collaborative groups which support those living with a rare disease. The aim of the survey was to help evaluate the impact of the COVID-19 pandemic on such groups by asking questions about the current services provided, changes in contact and demand for services, how they as a group have adapted to the new demands, and the longer term implications they anticipate for their group. Survey results may be used to help improve the accessibility of resources and address the needs of individuals affected by rare disease(s). The results may also identify strategies that are working well for these groups, as well as common issues which could be explored, such as where the development of further resources would be most effective during this COVID-19 pandemic.

## Data description

An online survey was constructed using an iterative approach with input from the Northern Ireland Rare Disease Partnership (NIRDP www.nirdp.org.uk), Health Research Charities Ireland (HRCI, http://hrci.ie/) and Rare Disease Ireland (RDI, http://rdi.ie/). Each section in the survey contained closed and open-ended questions and were displayed with the option to save and return at a later date. The survey was uploaded onto SmartSurvey (www.smartsurvey.co.uk), and shared on the associated social media pages and websites of the above mentioned groups as well as on the Queen’s University Belfast (QUB) rare disease website (https://www.qub.ac.uk/sites/RareDisease/). Adult representatives from any group supporting people with rare diseases were eligible to complete the survey which was open from mid-July until mid-September 2020. Rare disease collaborative groups who have been involved in research conducted by the Rare Disease Team at QUB previously, and provided consent, were emailed directly and invited to participate in this study. Ethical approval was provided by the Faculty of Medicine, Life and Health Sciences, QUB research ethics committee (MHLS 20_72). Data file 1 displays the summary information from the survey responses and Table 1 provides an overview of the data files/data sets.

**Table 1:** Overview of data files/data sets

Label	Name of data file/data set	File types (file extension)	Data repository and identifier (DOI or accession number)
Data file 1	Summary data from SmartSurvey output	PDF	OPENICPSR, Covid 19 Data Repository https://doi.org/10.3886/E127461V1-70940
Data file 2	DF2 Common concerns shared on helpline	MS Word file (.docx)	OPENICPSR, Covid 19 Data Repository https://doi.org/10.3886/E127461V1-70941
Data file 3	DF3 Positive and negative impacts on groups	MS Word file (.docx)	OPENICPSR, Covid 19 Data Repository https://doi.org/10.3886/E127461V1-70942
Data set 4	DF4 Help for the future requested by the groups	MS Word file (.docx)	OPENICPSR, Covid 19 Data Repository https://doi.org/10.3886/E127461V1-70943

Six rare disease individual or collaborative groups completed the online survey. The groups were established between 1963 to 2010 and operate within Northern Ireland, Republic of Ireland or United Kingdom wide.

### Changes to helpline

83% (n = 5) of groups have a helpline for support and advice. 33% (n = 2) said the number of calls to their helpline have significantly increased during the pandemic. The most common questions/issues received on the helpline during the month previous to the survey are displayed in data file 2. 50% (n = 3) of contact has been from those with a rare disease, followed by 33% (n = 2) from family members.

### Changes to communication

60% (n = 3) of groups have moved to online communication due to restrictions. Hosting virtual committee meetings, conducting zoom chats with members, issuing newsletters, and hosting weekly webinars have all become a regular part of the services provided by groups. Data file 3 displays the most lasting positive and negative impacts from COVID-19 pandemic on the groups and people supported, many of which are a result of the change in communication.

### Online presence

From 1st February 2020 to the end of May 2020 33% (n = 2) of groups experienced a significant increase (more than doubled) in the number of followers on their websites and social media feeds. 33% (n = 2) also saw a significant increase (more than doubled) in the number of direct requests for support/information via their website or social media.

### Financial impact

Two groups have seen a significant reduction in their fundraising for research activities during the pandemic with one group no longer able to conduct this activity. 40% (n = 2) of groups said their fundraising activities and/or income had been majorly impacted by COVID-19. None of the groups surveyed had received any Government support during the pandemic. There was a general concern expressed regarding fundraising and the longer term implications, ‘*Not sure if this can be sustained’.* Data file 4 displays the needs of groups in order for them to be supported in the longer term, including financial support.

It is hoped this study has highlighted the support individual and collaborative groups require and has raised awareness of their value. Additionally, the findings of this study could be of value to future studies conducted in this field.

## Limitations

- Only six groups responded to this survey, which may reflect the lack of time and resources available to many rare disease charities.
- It is acknowledged that the small number of respondents limits the transferability of the results. The survey was promoted online via rare disease local charities and QUB; other methods of dissemination may have helped to generate more respondents.
- The time constraints associated with the rapidly evolving COVID-19 restrictions meant the questions were only applicable for a short period of time.
- It is possible that low staffing issues, due to current circumstances such as furlough, meant the survey was missed by many groups. Email responses from contacts within these groups often included the phrase, ‘I am currently on furlough’. Resources are stretched at this time and many groups do not have the capacity to take part in activities beyond their basic duties.
- Although the use of an ‘online’ survey has many advantages, it could also be a limitation for those who do not use online platforms and who are not familiar with browsing the internet.
- The survey was only available in the English language; therefore it is possible that this may have introduced bias to the sample in that potential respondents may have been excluded.

## Data Availability

The data described in this Data note can be freely and openly accessed on *OPENICPSR, Covid 19 Data Repository* under https://doi.org/10.3886/E127461V1 [9]. Please see Table 1 for details and links to the data.

## Abbreviations

HRCI: Health Research Charities Ireland
NIRDP: Northern Ireland Rare Disease Partnership
QUB: Queen’s University, Belfast
RDI: Rare Disease Ireland
